# Supplementary material for: All-optical synthesis of an arbitrary linear transformation using diffractive surfaces
Source: Light Sci Appl. 2021 Sep 24;10:196. doi: 10.1038/s41377-021-00623-5 (PMC8463717; doi:10.1038/s41377-021-00623-5)
Supplement: Supplementary file 1 — Supplementary Information [file 41377_2021_623_MOESM1_ESM.pdf]

Supplementary Information for

All-Optical Synthesis of an Arbitrary Linear Transformation Using Diffractive Surfaces

Onur Kulce[1,2,3], Deniz Mengu[1,2,3], Yair Rivenson[1,2,3] , Aydogan Ozcan[1,2,3,*]

[1] Electrical and Computer Engineering Department, University of California, Los Angeles, CA, 90095, USA

[2] Bioengineering Department, University of California, Los Angeles, CA, 90095, USA

[3] California NanoSystems Institute, University of California, Los Angeles, CA, 90095, USA

* Corresponding author: ozcan@ucla.edu

Onur Kulce: onurkulce@ucla.edu

Deniz Mengu: denizmengu@ucla.edu

Yair Rivenson: rivensonyair@ucla.edu

Aydogan Ozcan: ozcan@ucla.edu

Telephone: +1 310-825-0915

Postal Address: 420 Westwood Plaza, UCLA, Los Angeles, CA, USA, 90095

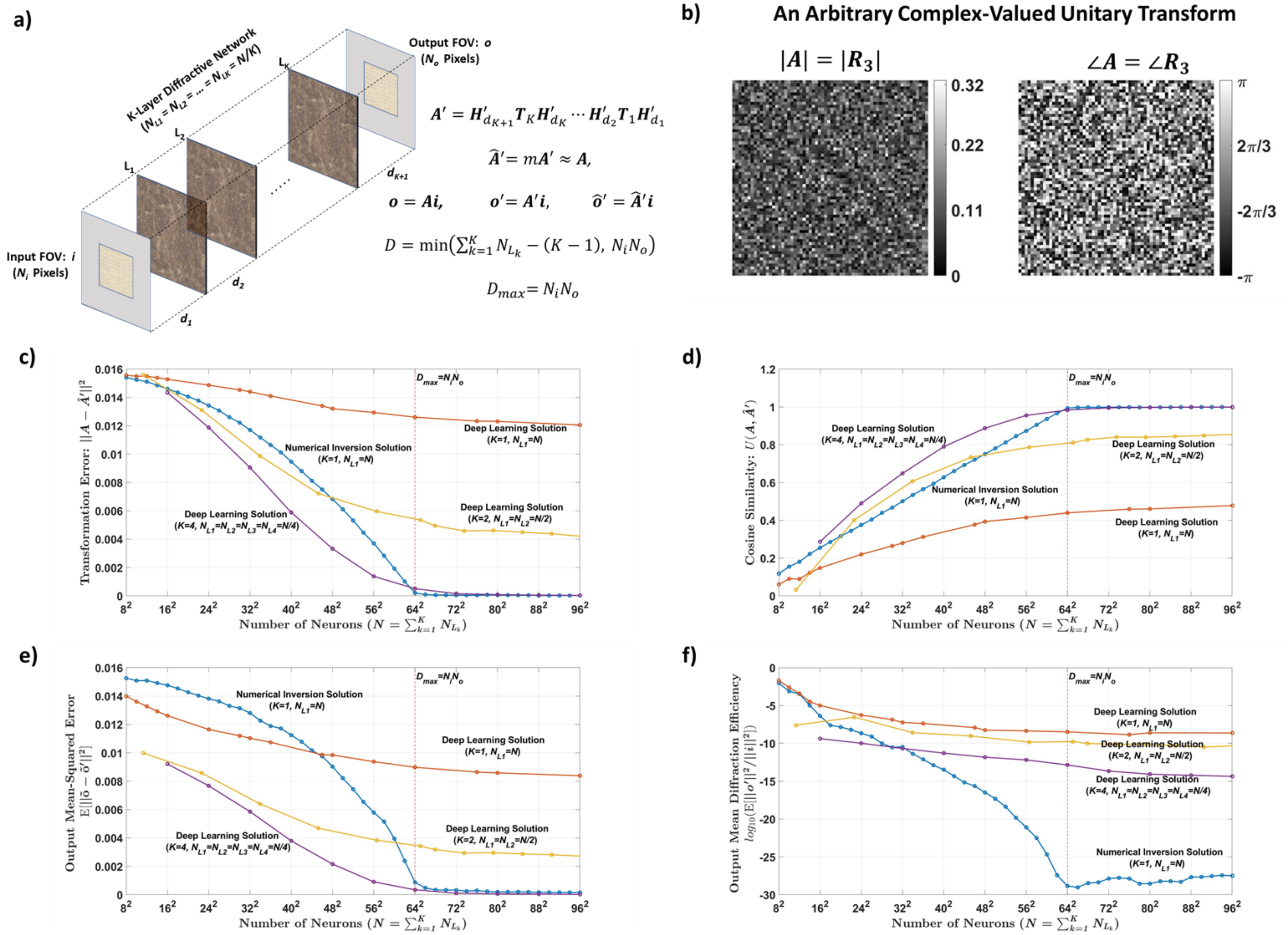

Figure S1: *Follows the caption of Fig. 1 (main text), except that the target ($A = R_3$) is a different, randomly generated unitary matrix, i.e., $R_3 \neq R_1$.*

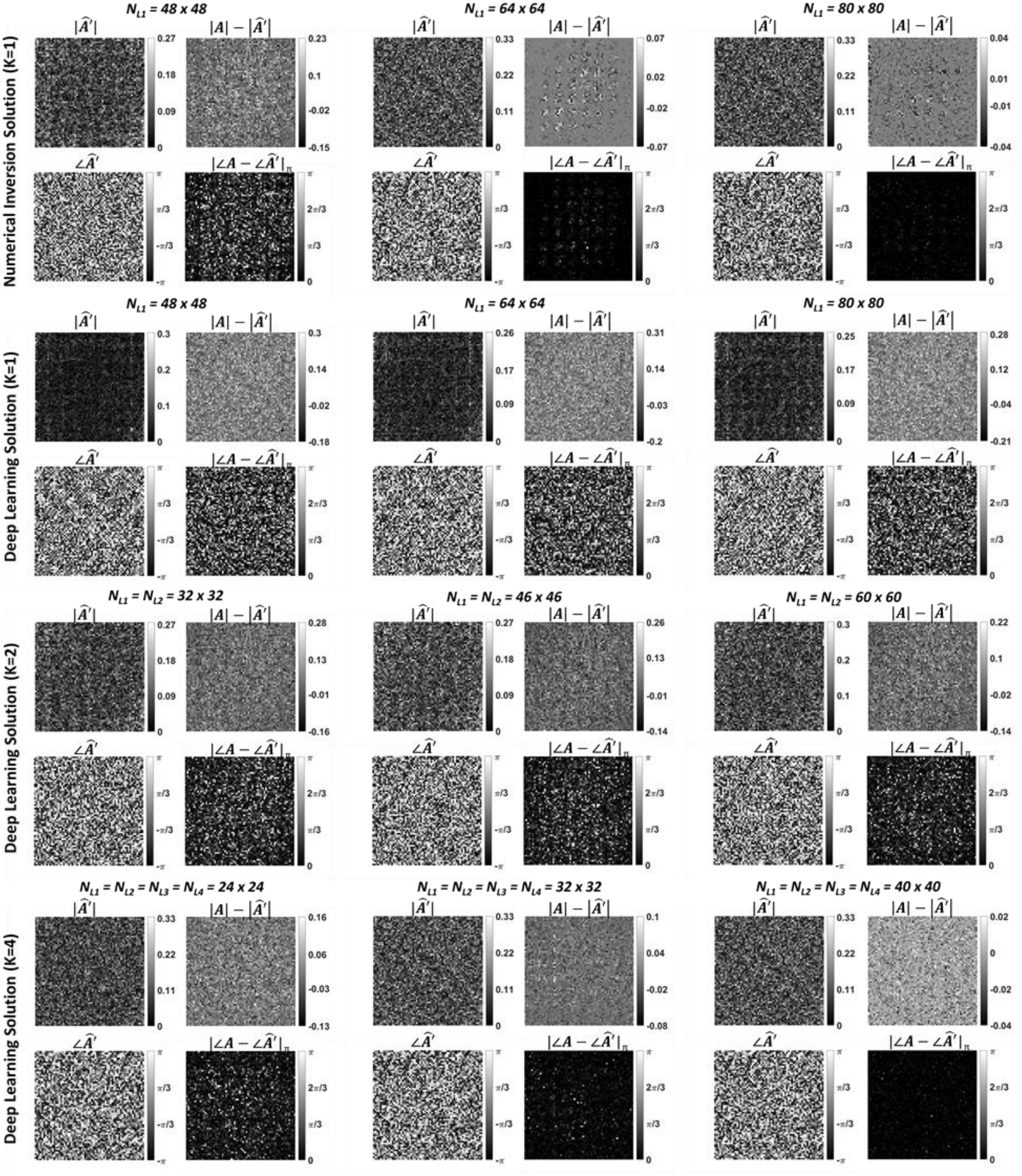

Figure S2: *Follows the caption of Fig. 2 (main text), except that the target ($A = R_3$) is a different, randomly generated unitary matrix, i.e.,* $R_3 \neq R_1$.

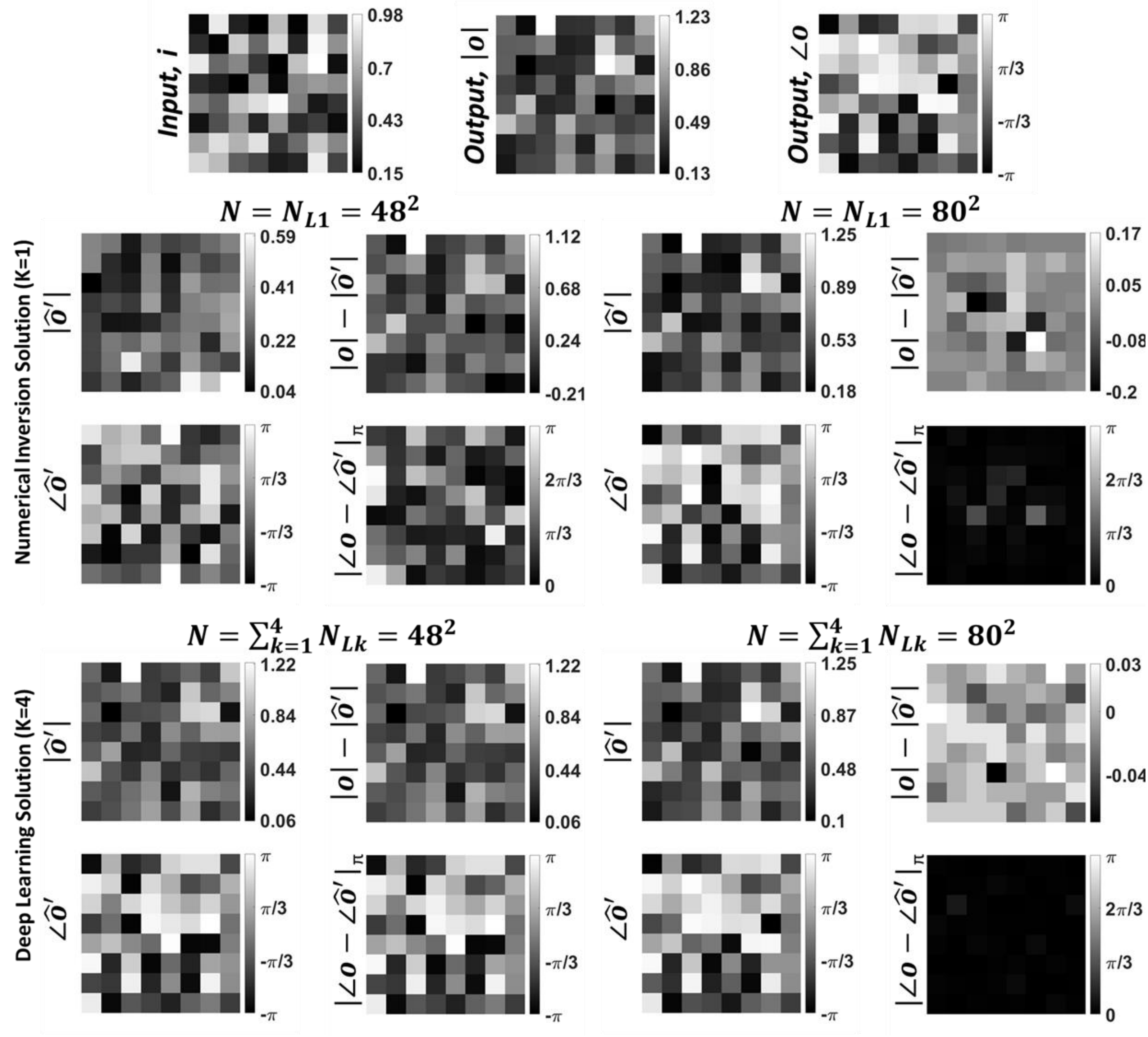

Figure S3: *Follows the caption of Fig. 3 (main text) except that the target* $(\boldsymbol{A} = \mathbf{R}_3)$ *is a different, randomly generated unitary matrix, i.e.,* $\mathbf{R}_3 \neq \boldsymbol{R}_1$.

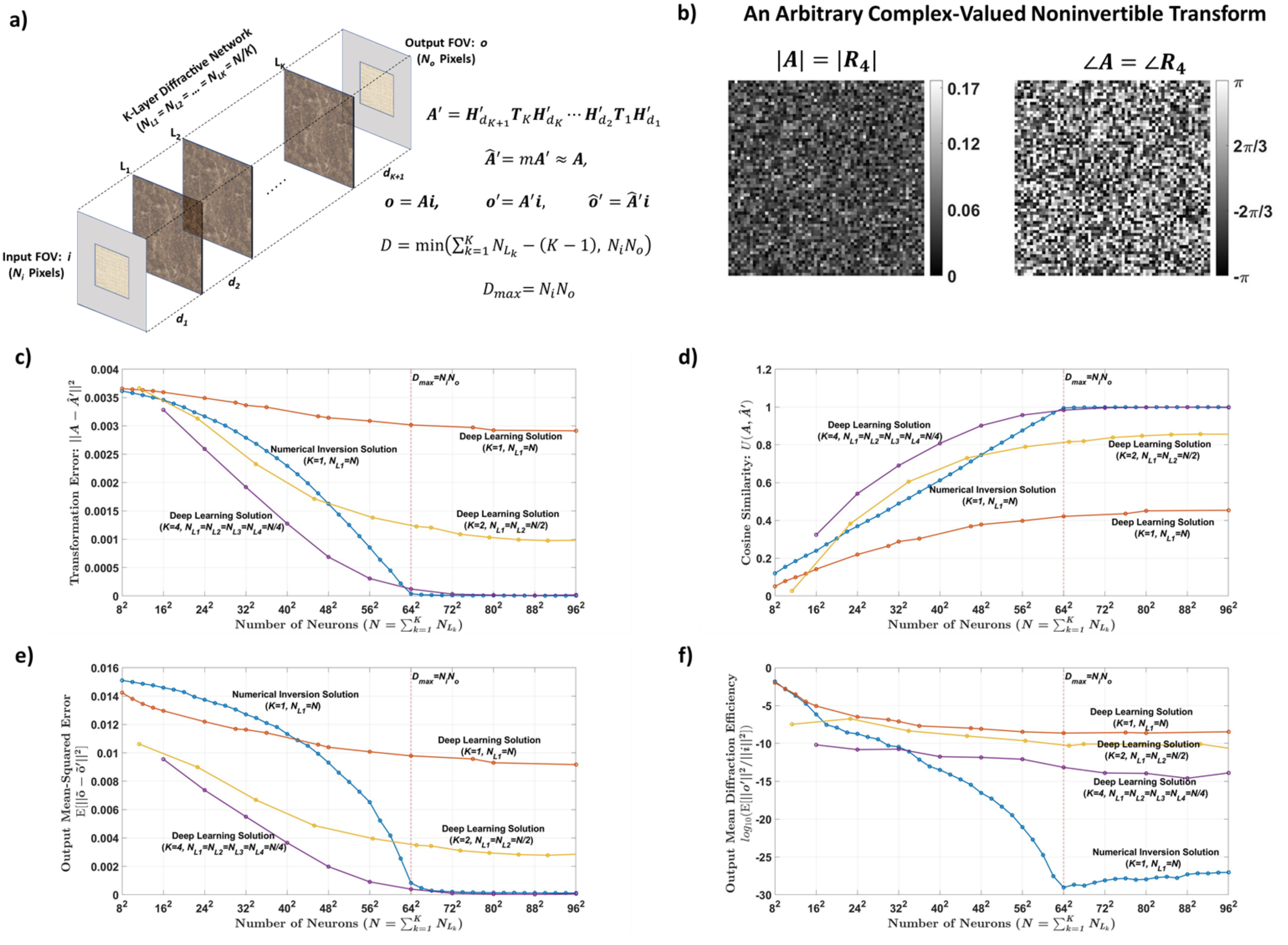

Figure S4: *Follows the caption of Fig. 1 (main text), except that the target* $(\boldsymbol{A} = \boldsymbol{R_4})$ *is a randomly generated noninvertible matrix.*

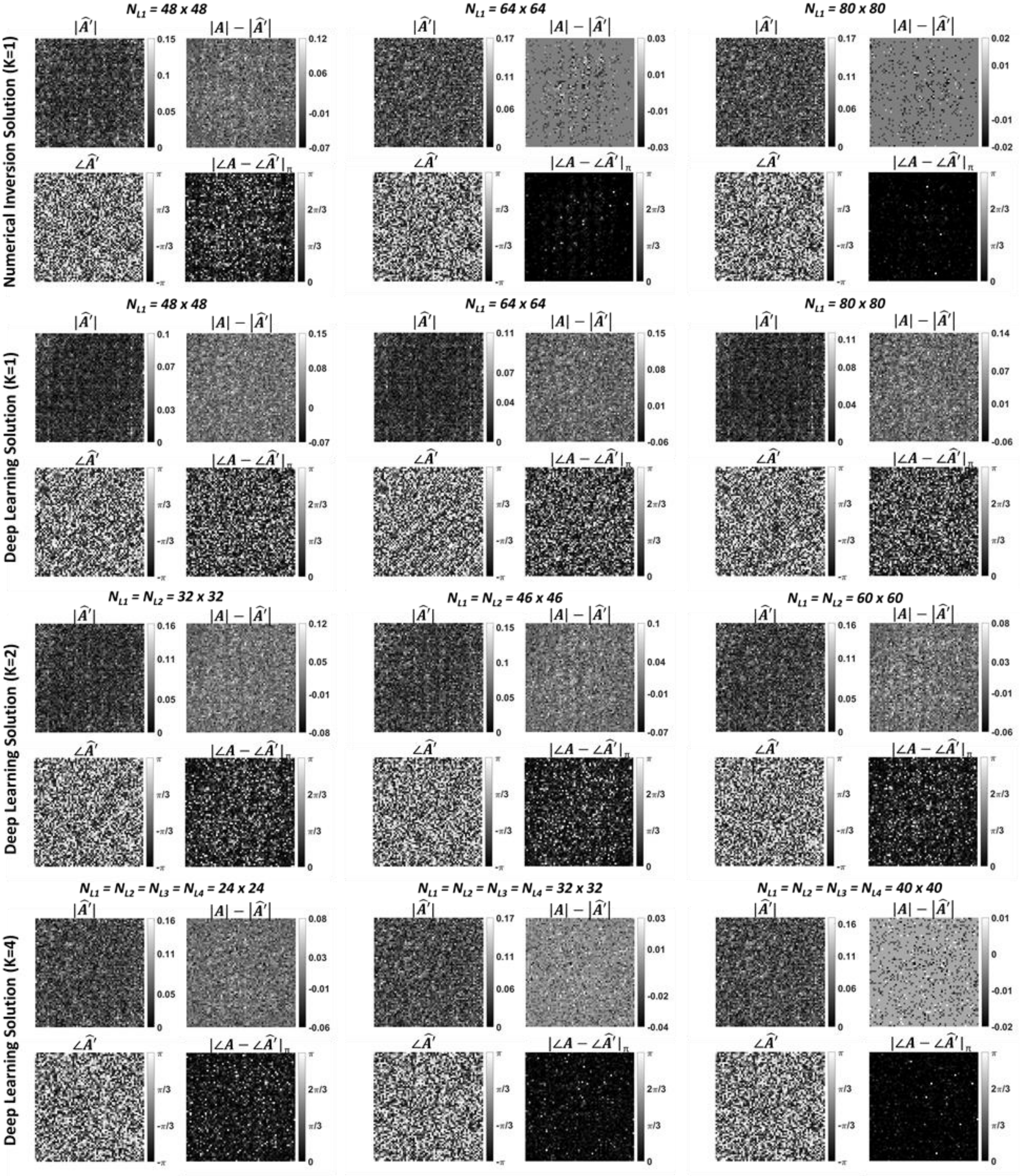

Figure S5: *Follows the caption of Fig. 2 (main text), except that the target* $(\boldsymbol{A} = \boldsymbol{R_4})$ *is a randomly generated noninvertible matrix.*

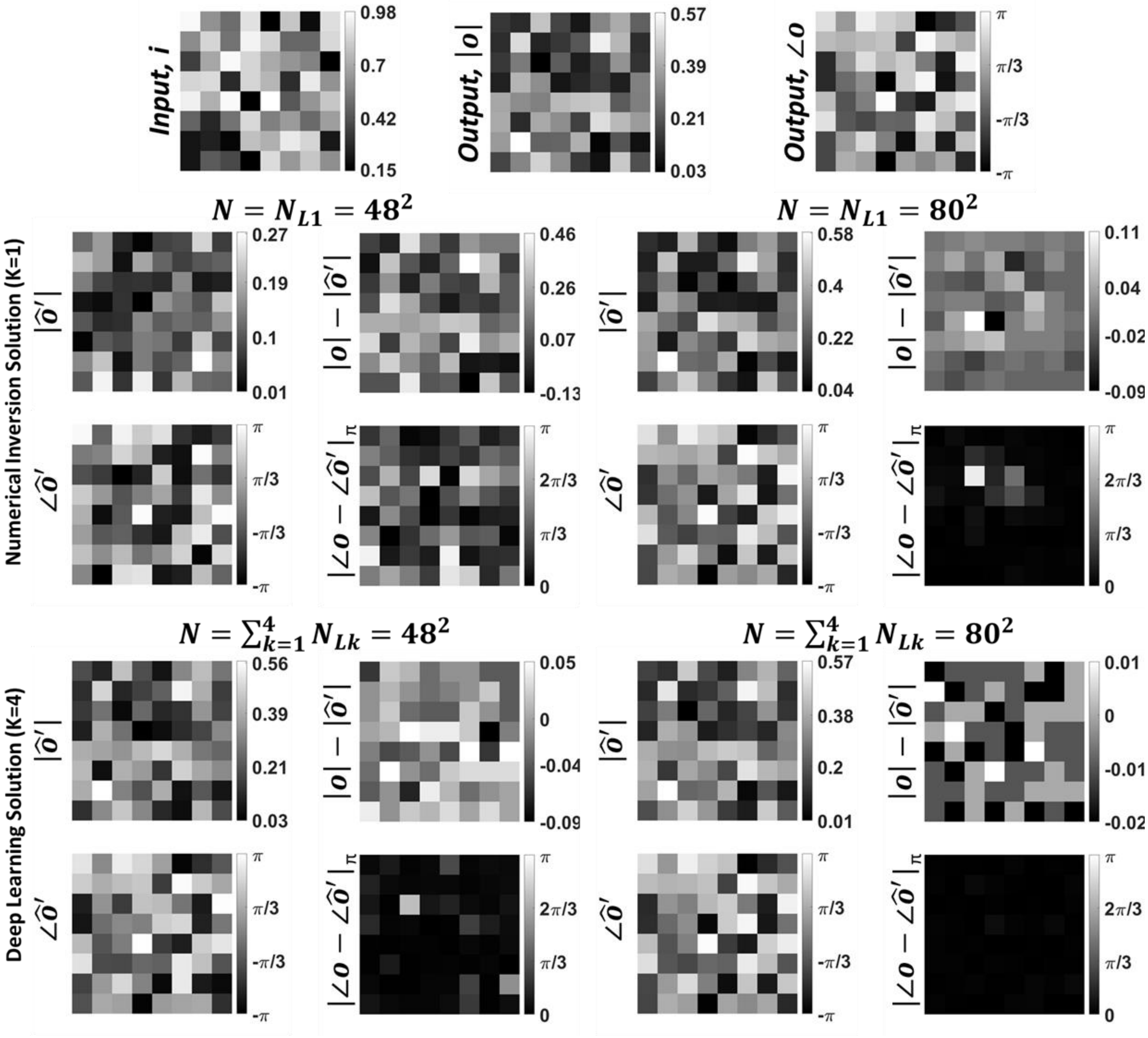

Figure S6: *Follows the caption of Fig. 3 (main text), except that the target* $(\boldsymbol{A} = \boldsymbol{R_4})$ *is a randomly generated noninvertible matrix.*

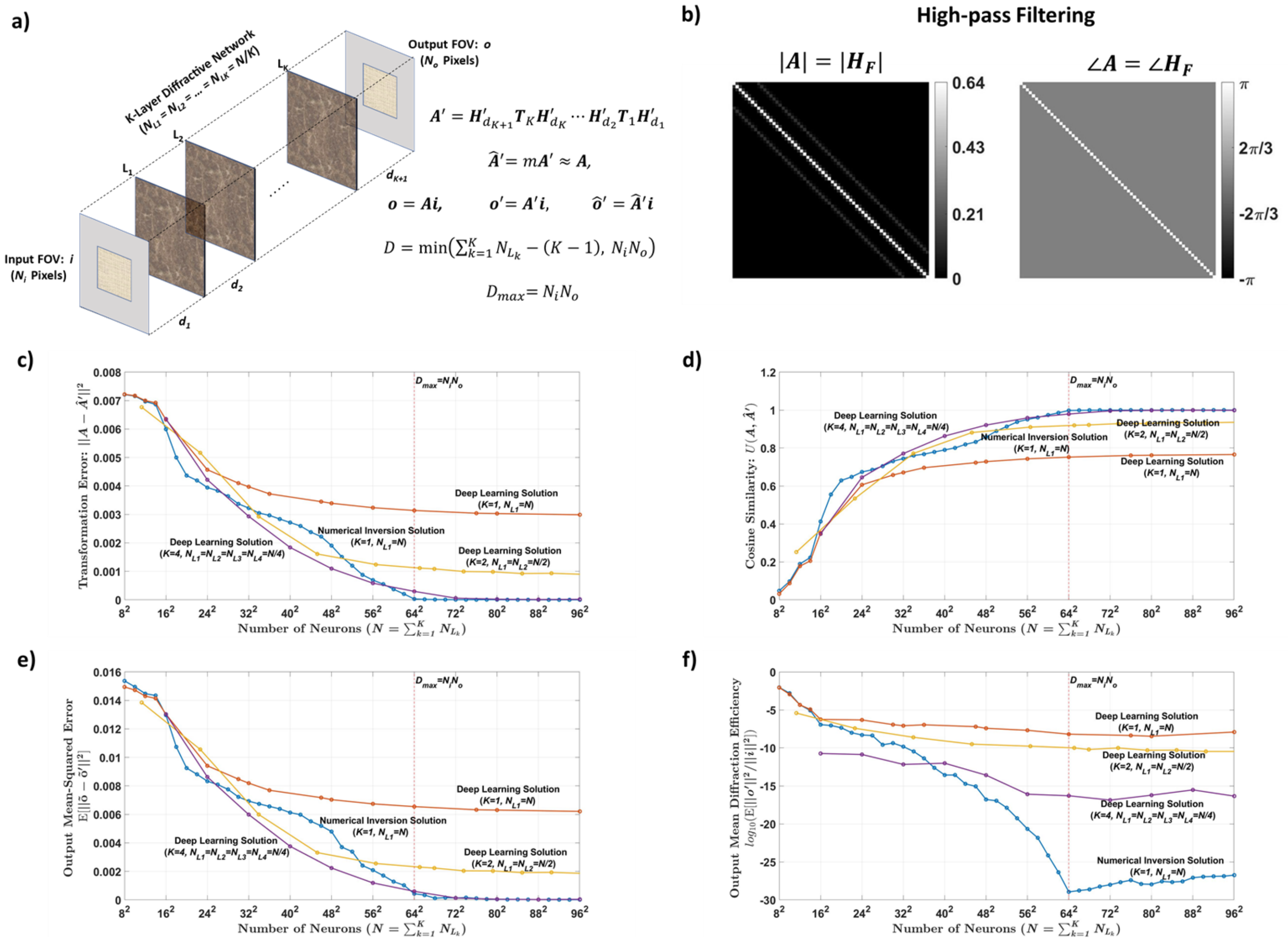

Figure S7: *Follows the caption of Fig. 1 (main text), except that the target ($A = H_F$) is a high-pass filtered coherent imaging operation.*

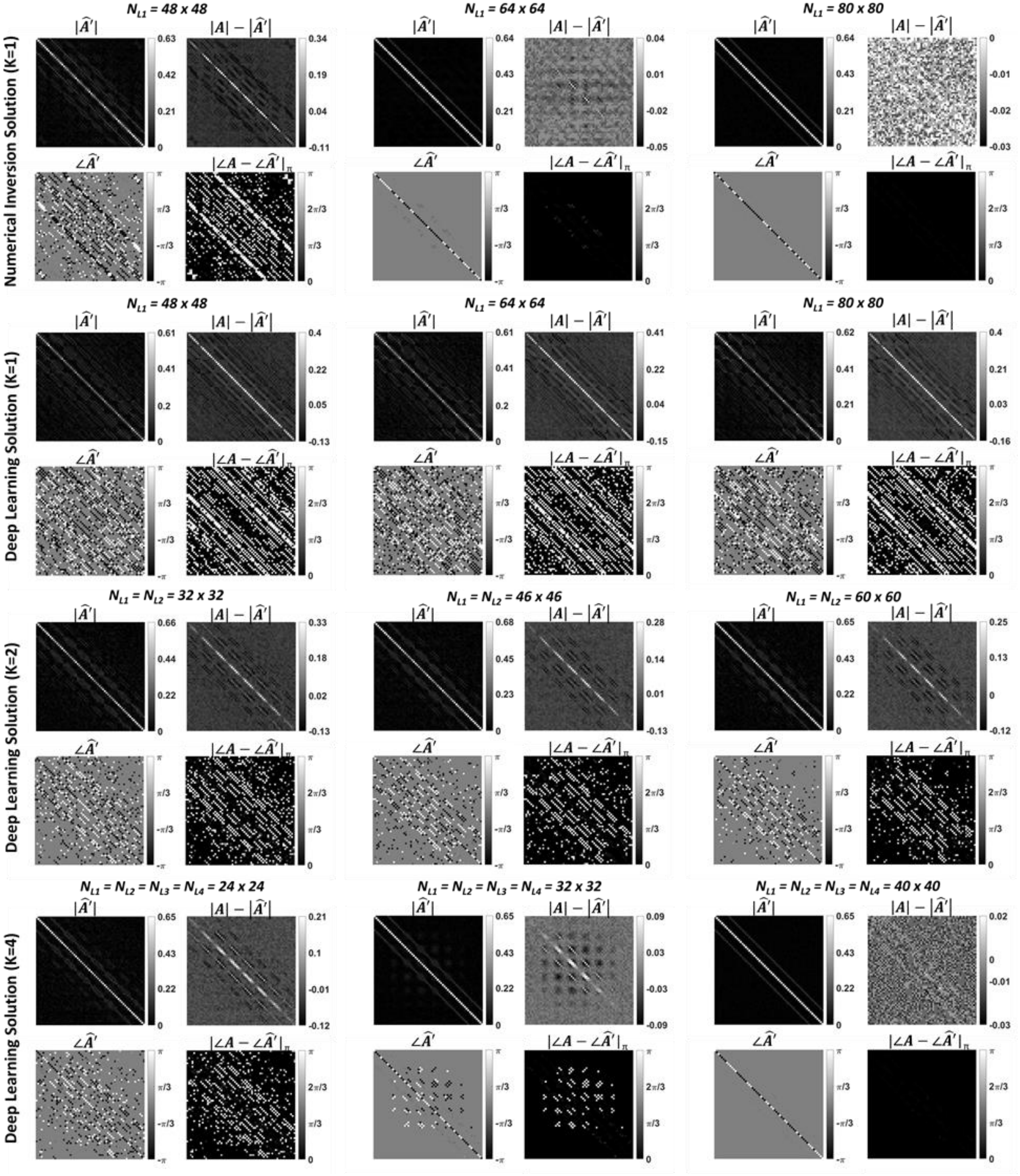

Figure S8: *Follows the caption of Fig. 2 (main text), except that the target* ($\boldsymbol{A} = \boldsymbol{H_F}$) *is a high-pass filtered coherent imaging operation.*

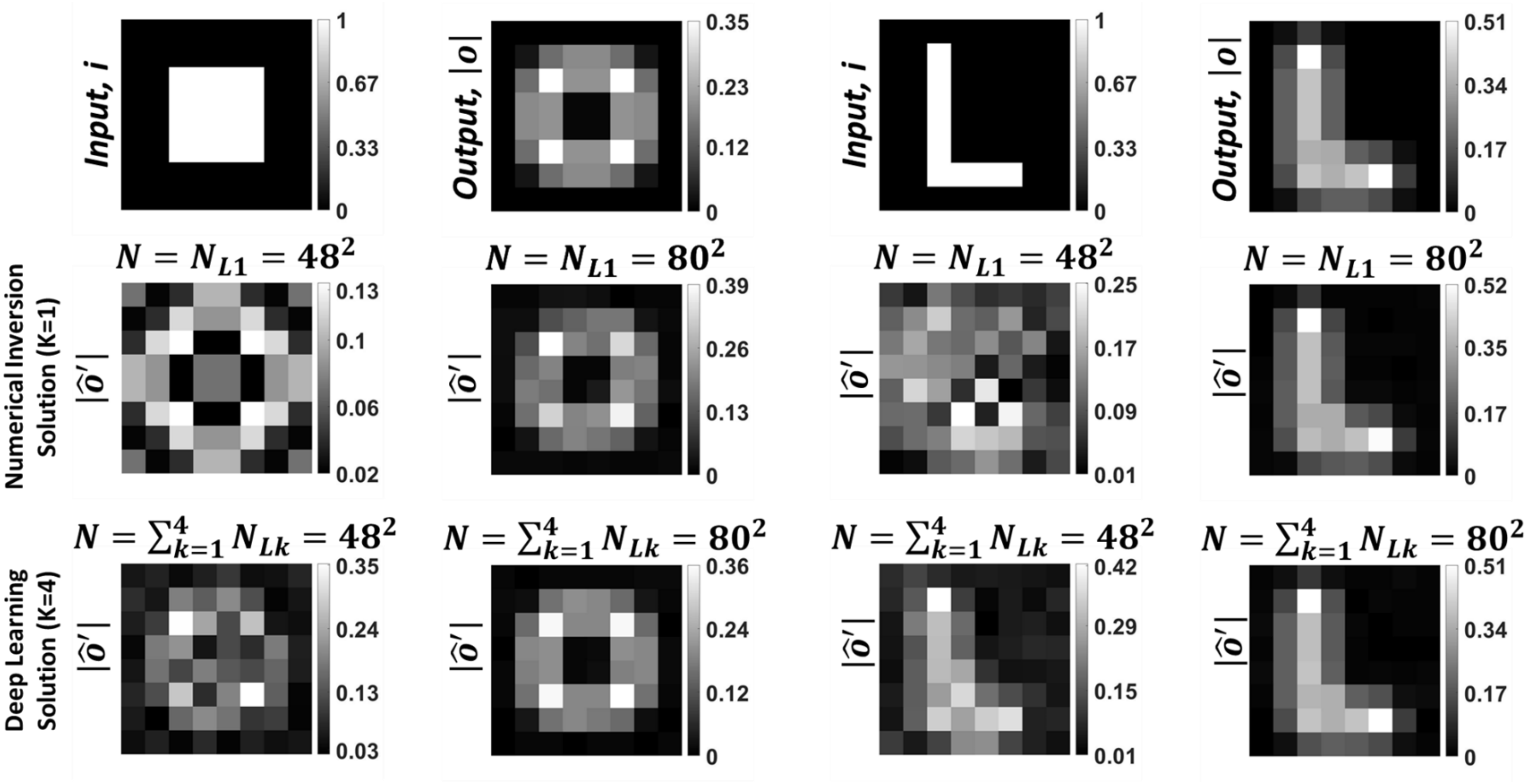

Figure S9: *Follows the caption of Fig. 3 (main text), except that the target* ($\boldsymbol{A} = \boldsymbol{H_F}$) *is a high-pass filtered coherent imaging operation.*

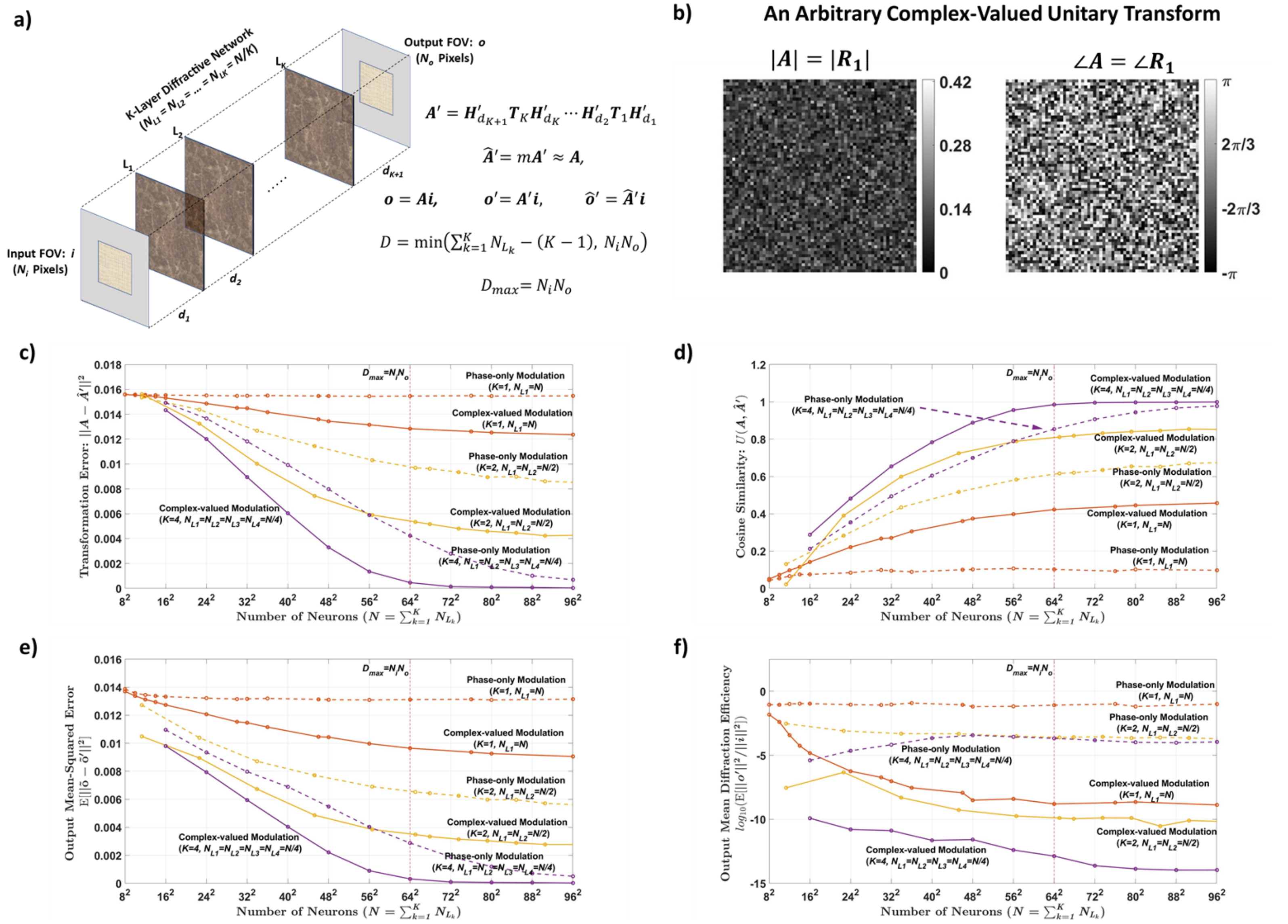

***Figure S10: Comparison of deep learning-based diffractive designs that use complex-valued vs. phase-only diffractive layers**. The target linear transformation shown in (b) is the same arbitrarily selected complex-valued unitary transform ($\boldsymbol{A} = \boldsymbol{R_1}$) reported in Fig. 1 (main text). In the complex-valued modulation scheme, both the magnitude and phase of the transmittance values of the diffractive neurons are optimized independently. In the phase-only modulation scheme, however, only the phase values are optimized while keeping the transmission magnitudes always 1. Figure caption follows Fig. 1 (main text).*

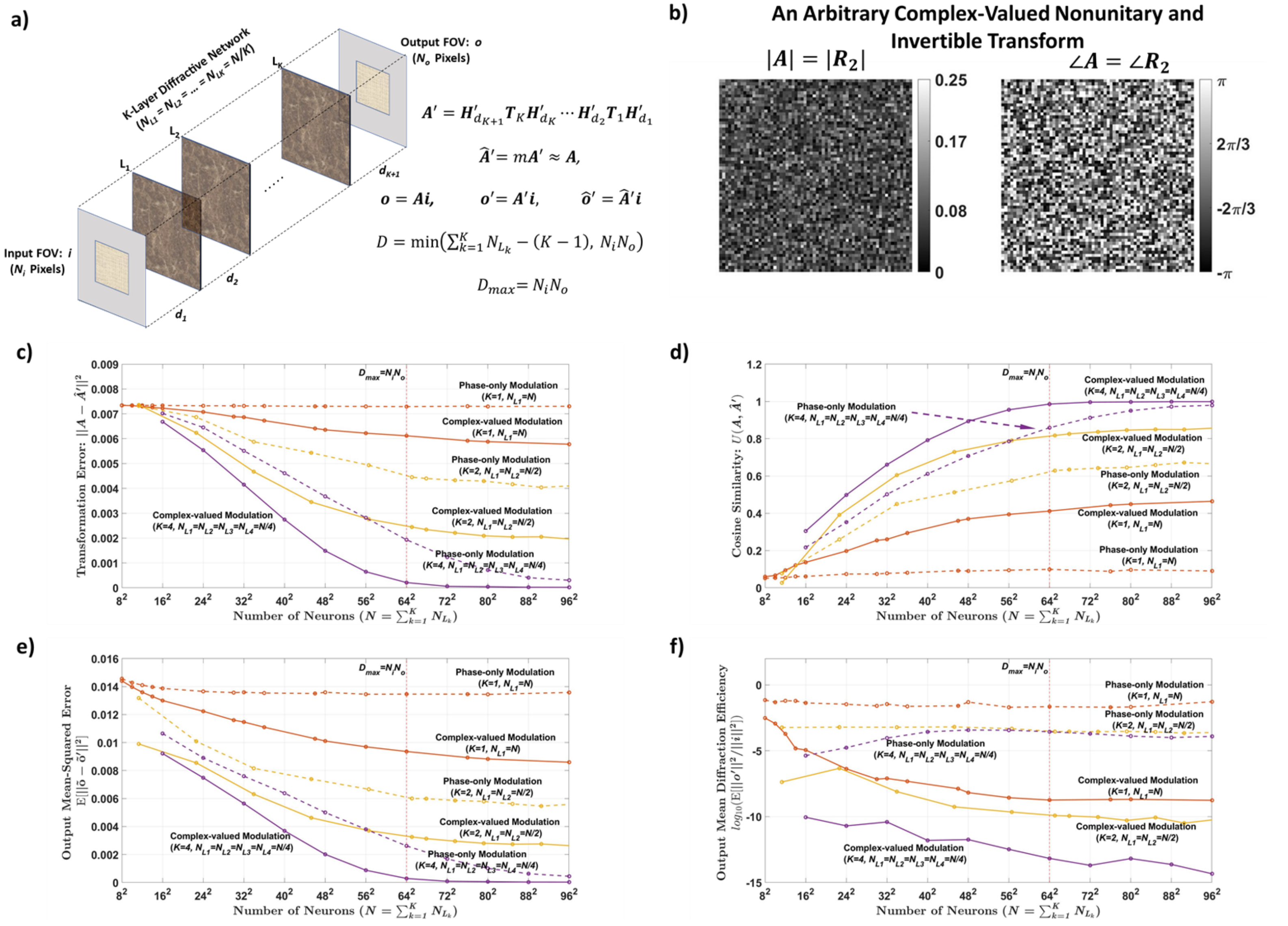

Figure S11: *Same as Supplementary Fig. S10, except for the arbitrary complex-valued nonunitary and invertible transform* $(\boldsymbol{A} = \boldsymbol{R_2})$ *shown in Fig. 4 (main text).*

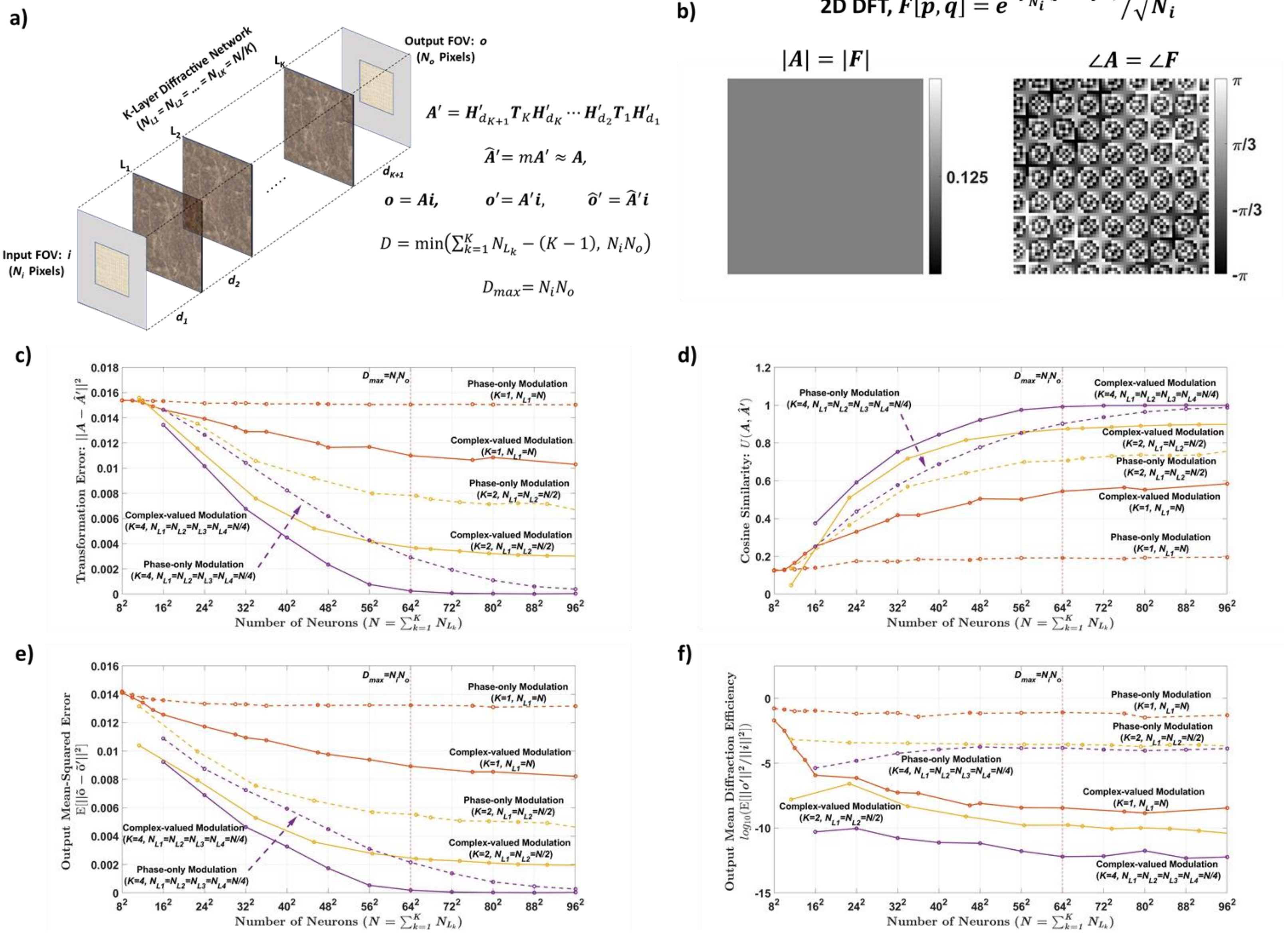

Figure S12: *Same as Supplementary Fig. S10, except for the 2D discrete Fourier transform* $(\boldsymbol{A} = \boldsymbol{F})$ *shown in Fig. 7 (main text).*

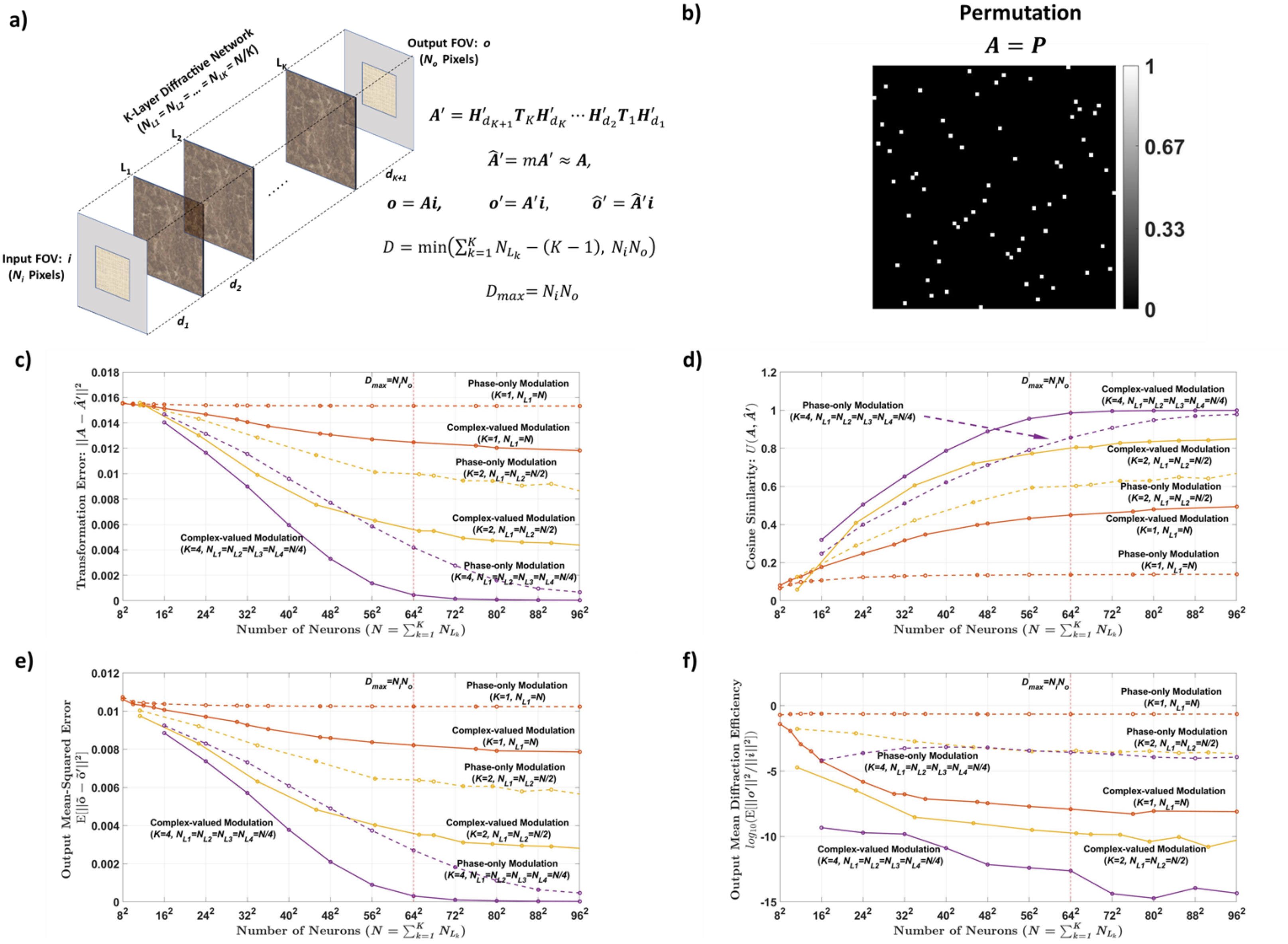

Figure S13: *Same as Supplementary Fig. S10, except for the randomly-selected permutation operation* ($\boldsymbol{A} = \boldsymbol{P}$) *shown in Fig. 10 (main text).*

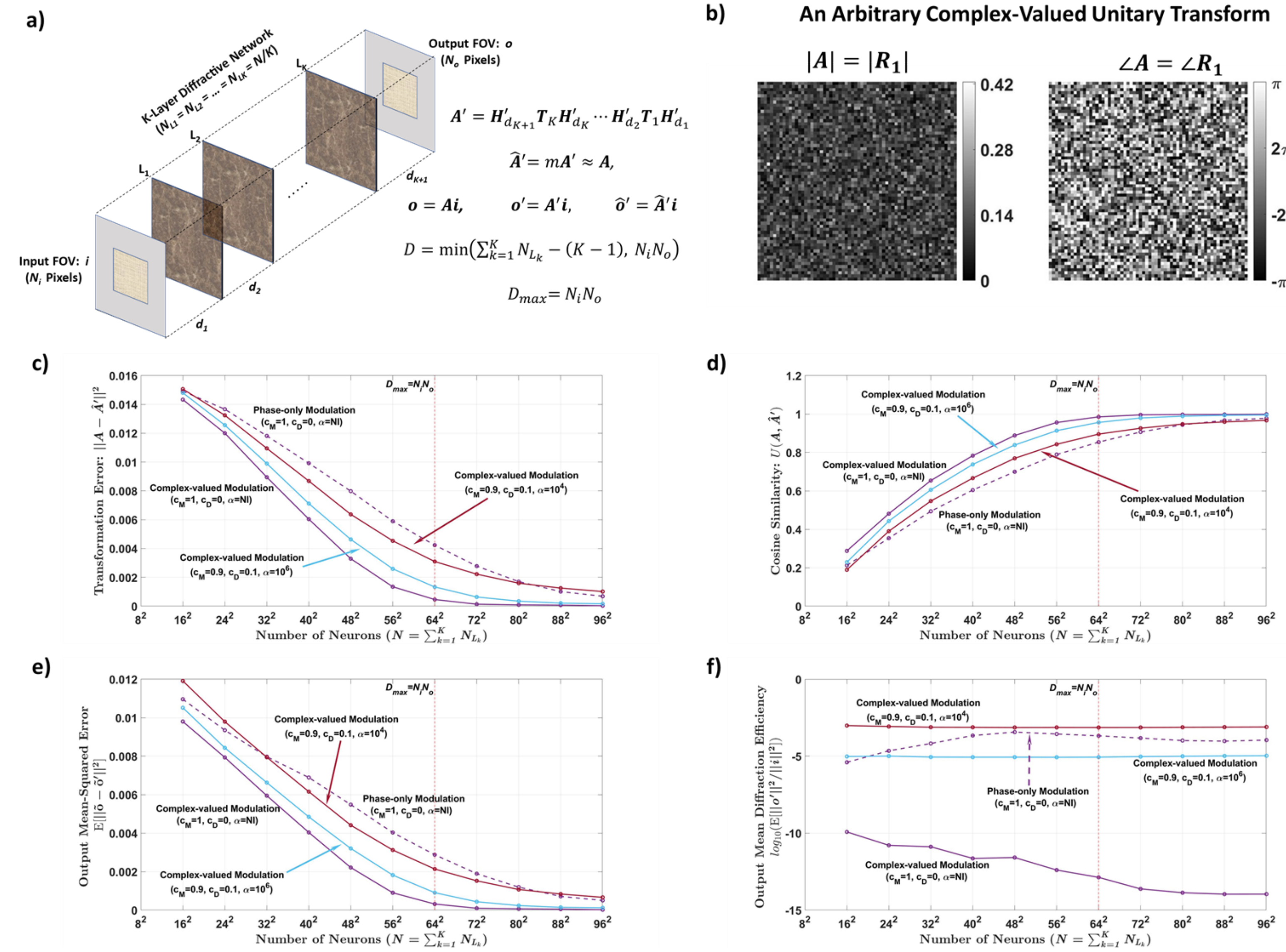

Figure S14: Comparison of different diffraction efficiency penalty parameters for 4-layer diffractive designs ($K = 4$). *The target linear transformation shown in (b) is the same arbitrarily-selected complex-valued unitary transform ($A = R_1$) shown in Fig. 1 (main text) and Supplementary Fig. S10. The* details of the diffraction efficiency loss and the parameters are presented in the Materials and Methods section and Equation 24 of main text. $\alpha = NI$ implies that the α value is "Not Important" when $c_D = 0$ (see Equations 24-25). Figure captions follow Fig. 1 (main text) and Supplementary Fig. S10.

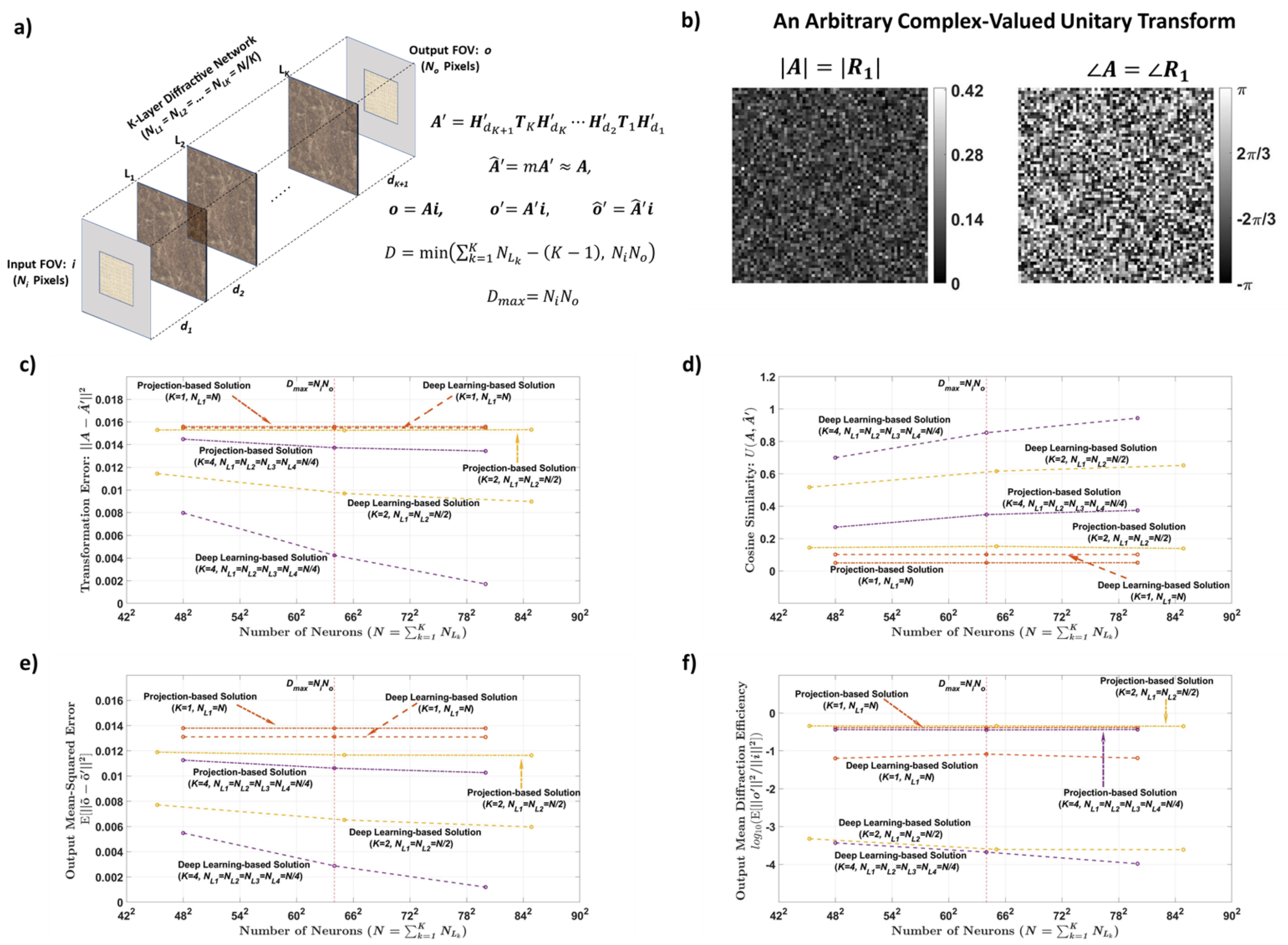

Figure S15: Comparison of phase-only diffractive designs based on deep learning vs. iterative projection-based solutions. *The target linear transformation shown in (b) is the same arbitrarily-selected complex-valued unitary transform* ($\boldsymbol{A} = \boldsymbol{R_1}$) *shown in Fig. 1 (main text) and Supplementary Fig. S10. Other details of the figure caption follow Fig. 1 (main text) and Supplementary Fig. S10.*

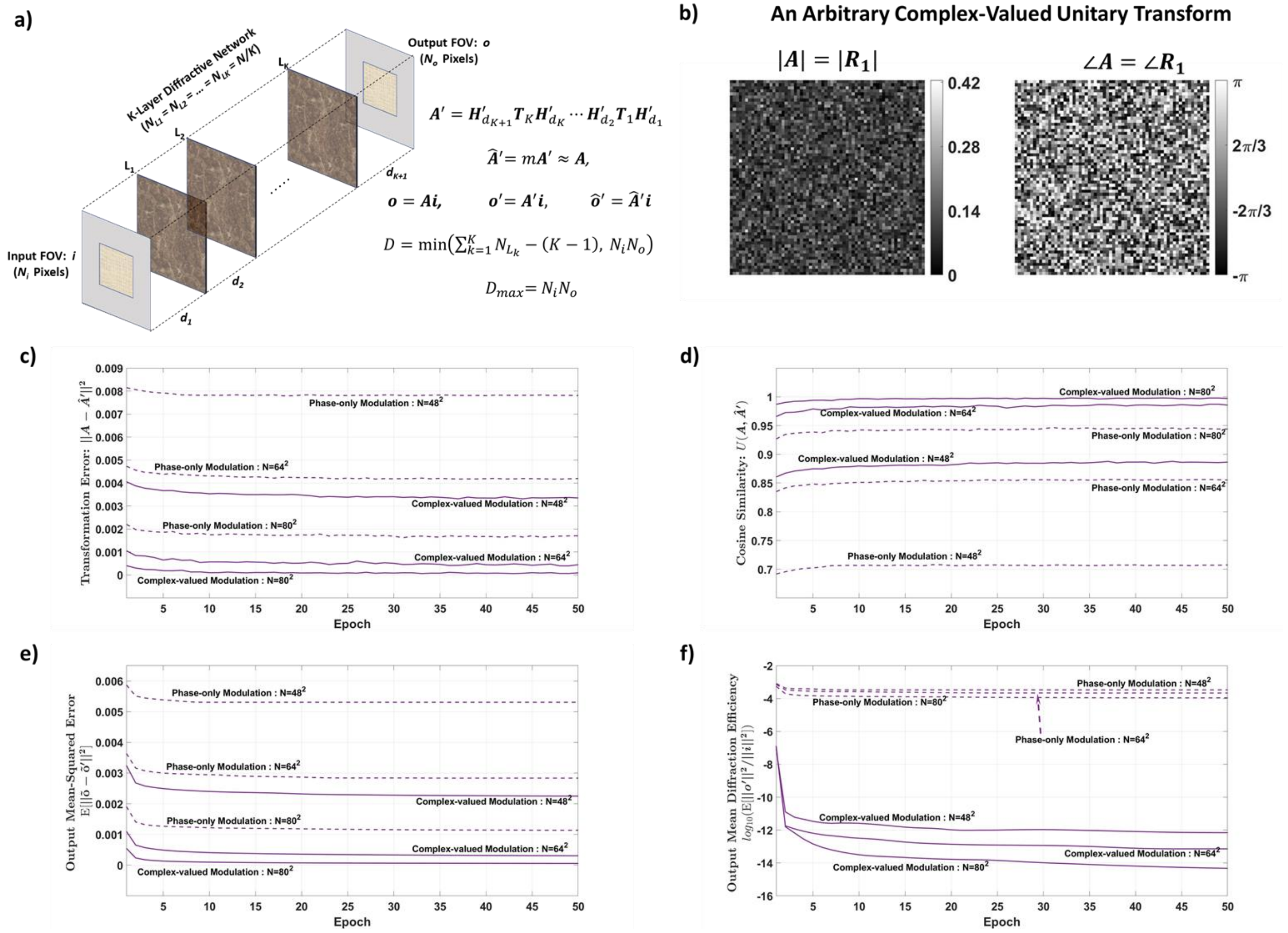

***Figure S16: Evolution of the performance metrics as a function of the number of training epochs and convergence of the deep learning-based algorithm for 4-layer diffractive designs ($K = 4$) with different number of total neurons**. The target linear transformation shown in (b) is the same arbitrarily-selected complex-valued unitary transform ($A = R_1$) shown in Fig. 1 (main text) and Supplementary Fig. S10. Figure caption follows Fig. 1 (main text) and Supplementary Fig. S10.*

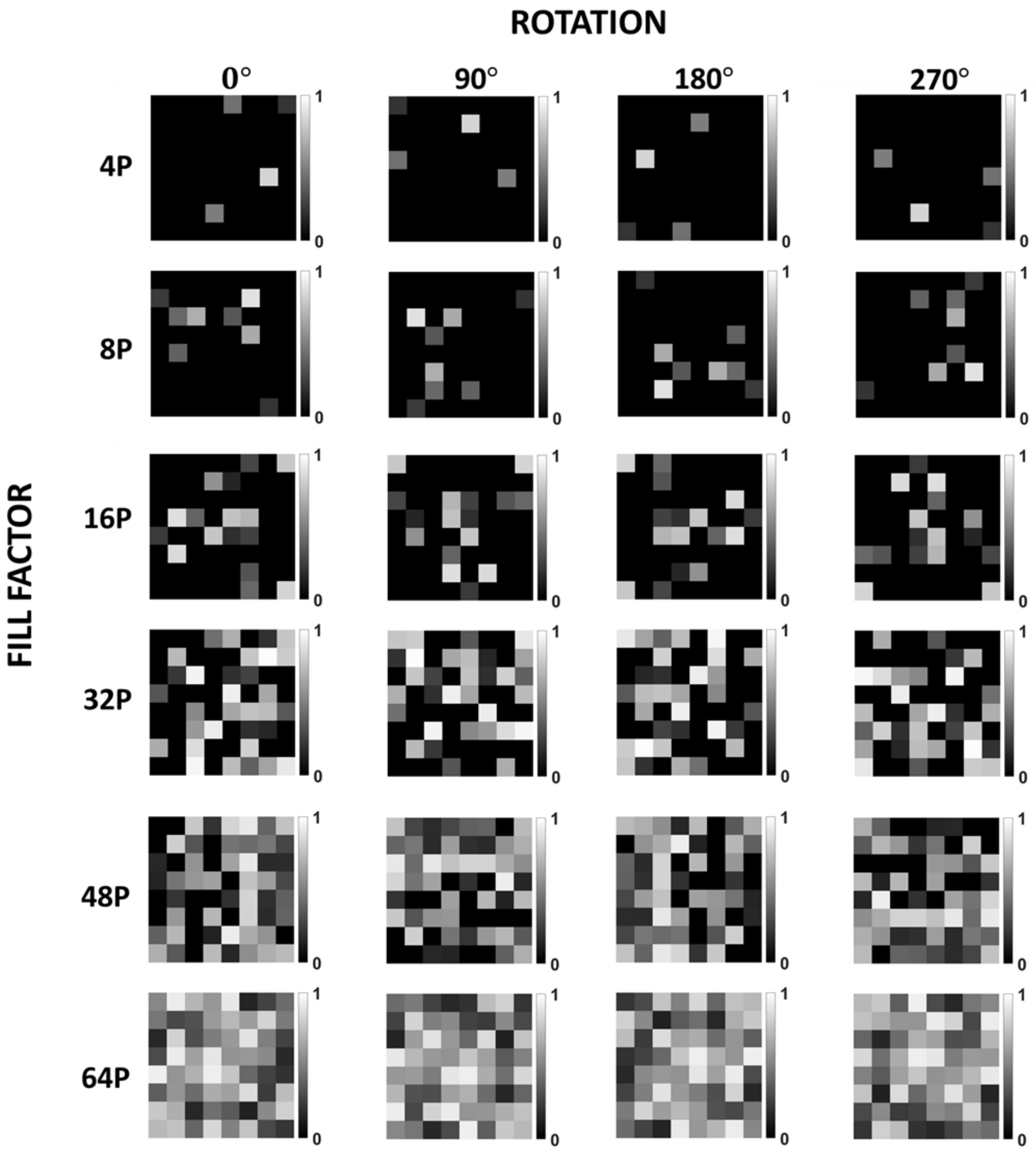

Figure S17: Sample images from the training input image dataset. *Each column indicates a different angle of image rotation. Each row indicates a different sparsity ratio or fill factor.* $16P$*, for example, indicates that 16 out of 64 pixels are nonzero. See Equation 15 of the main text for the details of the image dataset generation.*
